# Examining the association between cultural self-construal and dream structures in the United States and Japan

**DOI:** 10.3389/fpsyg.2023.1069406

**Published:** 2023-02-17

**Authors:** Hisae Konakawa, Toshio Kawai, Yasuhiro Tanaka, Chihiro Hatanaka, Kimberly Bowen, Alethea Koh

**Affiliations:** ^1^Uehiro Research Division, Institute for the Future of Human Society, Kyoto University, Kyoto, Japan; ^2^Institute for the Future of Human Society, Kyoto University, Kyoto, Japan; ^3^Graduate School of Education, Kyoto University, Kyoto, Japan

**Keywords:** culture, self-construal, independence-interdependence, dreams, empirical dream research, analytic and holistic thinking

## Abstract

Cultural differences in self-construal, human relationships, and values between Western and East Asian people have been suggested. The aim of this article is to investigate cultural difference in dreamers’ self-construal based on their dreams. We examined the dreams sampled via online questionnaires from 300 non-clinical participants from America and Japan, respectively. The free response for the contents of “impressive dreams in childhood” “recent impressive dreams” was categorized into the five general dream structural patterns. Besides, the participants were asked to answer the scales to investigate participants’ cultural self-construal. The current results revealed the prevalence of the independent view of self in American participants and the interdependent view of self in Japanese participants. In addition, we found significant cultural differences in the dream length and structural patterns. For American dreams, the dream-ego had a clear will and strong mobility, and there were obvious ends of dream events. Conversely, for Japanese dreams, the weak agency and vague conscious of the dream-ego were shown, and others could play a main role in one’s dreams. These results suggested that each characteristic of the American and Japanese samples may be influenced by the differences in self-construal or in the process of self-formation between American and Japanese cultures.

## Introduction

This current research focuses on examining cultural differences in self-construal through agency in dream content between America and Japan. To elucidate the self-construal in dream content, we regard the dream-ego as the protagonist in the dream event while focusing on dream structures. This current study is novel in the focus on dream structures, as previous research on dreams has mainly focused on symbolism or individual case studies. To examine dream structures, we also regard dreams as narratives and use “Structural Dream Analysis (Roesler, 2018a,b)” which adopts two methods for the analysis of narratives (Propp, 1975; Boothe, 1994).

Structural Dream Analysis is constituted of general structural patterns with subclassifications, which are made up based on the dream ego’s initiative in relationships with others or capacity to deal with challenges in dream events. Hence, the subclassifications capture the dreamer’s self-construal through agency in dream content. The detailed explanation of Structural Dream Analysis is presented in the method section below. Structural Dream Analysis originally aims to discuss common functions of dreams from the psychotherapeutic perspective and apply it to daily life for the non-clinical population. It focuses on the development and maturity of human’s psyche (Roesler, 2018a,b) by identifying similarity between individuals’ dream series as the dreamers’ conditions improve through psychotherapy without other factors such as dreamers’ psychological symptoms. Therefore, it has not only been used for within-subject comparisons, also been shown to be suitable for between-group comparisons. In a previous study on dreams of persons from Germany and Japan being in psychotherapy, Structural Dream Analysis revealed cultural differences in dreams between German and Japanese samples (Roesler et al., 2021). The current study increased the number of samples and compared dreams between America and Japan by Structural Dream Analysis to reveal the connection between dreams and cultural construal.

Previous studies dealing with non-clinical populations have revealed cultural differences in human relationships and values between Western and East Asian people.

One such cultural difference is found in self-construal, which refers to individuals’ own conceptualization of who they are, or how they experience themselves to be (Kitayama, 1994). Many psychological processes such as thinking styles, emotion, and motivation have been shown to change in response to one’s self-construal (Cross et al., 2002; Shao et al., 2018). Particularly, self-construal described as interdependent or independent views of self, is commonly studied in comparisons between the West and East as representatives of each type of culture. Aspects of one’s culture, such as manners, language, customs, norms, and social systems, foster culturally-specific self-construal due to the values and priorities made salient to the individual, which may unconsciously shape individuals’ perception and definitions of their selves. In the West such as the US, the independent self is defined by internal attributes which is less influenced by others and their surrounding environment. Whereas in East Asia such as Japan, the interdependent self is defined in relation to others and their surrounding environment, where the context is the dominant influence (Markus and Kitayama, 1991). These cultures also show general differences in cognitive set or style between analytic thinking and holistic thinking. For example, Americans tend to use an either/or approach style, and value individuality while Japanese prefer a harmonious style, and value the maintenance of social relations (Nisbett, 2003).

In addition, research in cultural products has shown that items produced by individuals (etc., stories, folklore, music) are influenced and shaped by the culture in which they are embedded. Cultural differences in self-construal are similarly reflected in fairy tales. Regarding Western and East Asian fairy tales, cultural differences in the protagonist’s behavior or narrative end have been observed (Kawai, 1995). For instance, unlike Western stories where the protagonist’s role is to fight and defeat the villain such as in the Grimm fairy tales, there is the ambiguity about the motivations of the protagonist or narrative end in Japanese fairy tales. Moreover, just as the individual creates the product, these products go on to reinforce cultural ideas within the consumers of the product. These cultural messages help shape individuals’ schema of how things work in the world, such as the relationship between the conscious and unconscious through the narrative format.

In a similar manner as the above cultural narratives, dream content is also embedded within cultural contexts. For instance, analytical psychology assumes that “dreams contribute to the self-regulation of the psyche by automatically bringing up everything that repressed or neglected or unknown” (Jung, 1952). The self in dreams is referred to as the dream-ego, which has been regarded as “the potential image of the ego” (Kawai, 1971). The dream-ego is differentiated from one’s actual self, being more like an actor representing the self within a dream. Given that the dream-ego has the agency to think within the dream, the dreamer’s values, beliefs, and cultural background could also shape how the dream-ego acts in the dream. Therefore, the actions and thoughts of the dream-ego in the dream may reflect dreamer’s self-construal, as in real life. However, there is a severe lack of focal research from this perspective and further empirical research is necessary to unravel the validity of this connection.

To date, there are few cross-cultural studies on dreams, and cultural differences in dreams remain unclear (DeCicco et al., 2012). Therefore, we are interested in examining the cultural differences between American and Japanese dreams, and studying the connection between independent and interdependent self-construal and dream structures in the current research.

We hypothesize that Americans and Japanese will, respectively, show differences in the subclassifications of Structural Dream Analysis. For example, American dreams will be more categorized into the subclassifications where the dream-ego is active and get initiative than Japanese. Because the behavior of the dream-ego will reflect cultural differences in participants’ self-construal regarding agency and centrality of self as the individual/bounded/focal actor. In that sense, this study offers further evidence that cultural milieu shapes the self both at the explicit and implicit levels, because dreams represent one of the most extreme examples of “implicit” psychological processes. For example, the individual differences in implicit learning were related to the incorporation of waking events into dreams (Wang et al., 2021), which give some evidence for the correlation between dream content and implicit learning process for waking-life experiences.

In addition, we are also looking at independent and interdependent views of self, the anthropophobia mentality, and the sense of self in a exploratory manner to investigate participants’ cultural self-construal in this study. The concept of “sense of self” is also measured to capture the degree of integration of participants’ self. By organizing and processing one’s psychological experience subjectively, the individual creates a unity called “self,” and the “sense of self” is defined as the subject responsible for this organizing action at a multilayered level such as through the senses, body, and language (Stern, 1985). Especially for the unique characteristics of the Japanese self, past research has been dedicated to studying the concept of the “anthropophobic mentality.” Anthropophobia is related to feeling afraid of neighbors and acquaintances (people in intermediate relationships) or feeling threatened by the gaze of other persons (Nagata, 1992). It has been a typical neurosis for Japanese. Japanese with interdependent self-construal tend to focus on fitting in and being accommodating to others, because these acts give rise to pleasant, other-focused emotions (e.g., feeling of connection) while diminishing unpleasant ones (e.g., shame; Markus and Kitayama, 1991). It specifically taps into the degree that participants give weight on others’ perspective to define their self. We predict that American and Japanese will, respectively, show differences in independent and interdependent self-construal in the anthropophobia mentality scale (Horii, 2012), the sense of self scale (Matsuoka, 2015), and the independent and interdependent view of self scale (Uchida, 2008). Similar to previous studies, we posit that Americans will score higher in independent self-construal and Japanese will score higher in interdependent self-construal.

## Materials and methods

### Study design

#### Participants

Three hundred non-clinical participants in America and Japan were sampled, respectively, through online platforms, Amazon Mechanical Turk in America and Lancers in Japan. During recruitment, participants were asked and filtered according to their age (above 18 years) and nationality. After removing incomplete responses, 220 samples (135 males, Mean age = 36.4 years, SD = 10.1) from America and 257 samples from Japan (146 males, Mean age = 39.9 years, SD = 10.6) remained. Participants were informed that they were free to answer or decline the questionnaire, and that anonymity was assured. All data were analyzed anonymously.

#### Procedure

The survey was conducted in June and August 2020 and was approved by the Ethical Review Committee for Clinical Psychology Research at Kyoto University. We recruited participants through online platforms: Amazon Mechanical Turk in America and Lancers in Japan. Participants signed up for a survey studying their daily experiences and dreams occurring during sleep. Participants were asked to click on the link provided by the online platforms to access the questionnaire. Participants’ demographics, target measures, and dream content were then collected in the order listed in the measures section below. Missing data were removed before coding *via* Structural Dream Analysis.

#### Measures

The questionnaire contained items concerning dreams, the anthropophobia mentality scale (Horii, 2012), the sense of self scale (Matsuoka, 2015), and the independent and interdependent view of self scale (Uchida, 2008). The anthropophobia mentality scale and the sense of self scale were translated from Japanese to English by fluent English speakers of the research team. In addition, the coding for this study was conducted by fluent English-speaking members of the research team for American samples and native Japanese speakers of the research team for Japanese samples. A reliability test found an interrater agreement for the results coming from the same case of *k* = 0.92. and disagreements in coding were resolved based on discussion.

##### Anthropophobia mentality

The anthropophobia mentality scale (Horii, 2012) consists of 30 items measured using a 7-point Likert-scale, from 0 (Definitely not applicable) to 6 (Highly applicable). These items covered six components of anthropophobia mentality: (I). Worries about oneself and others (e.g., “Become very anxious about how others think of me”), (II). Worries that cannot fit into the group (e.g., “Cannot fit in well with a group”), (III). Worries in social situations (e.g., “Feel intimidated in front of others”), (IV). Worries about eye contact (e.g., “Cannot make eye contact with others”), (V). Worries that cannot control oneself (e.g., “Give up easily due to lack of patience”), and (VI). Worries that be tired of living (e.g., “Cannot find meaning in life”). The contents of the anthropophobia mentality scale strongly reflect participants’ weight of emphasis and concerns about social interaction, which is a strong factor contributing to differences between interdependent and independent self-construal (Kitayama et al., 2006; Uchida et al., 2009). The internal consistency of the scale was high in the present study (Cronbach’s *α* = 0.98).

##### Sense of self

The sense of self scale (Matsuoka, 2015) consists of 23 items measured using a 5-point Likert-scale, from 1 (Not at all) to 5 (Frequently). These items covered four components of the sense of self: Sense of Core Self (e.g., “Unsure of the range of my body”), Sense of Emotion Self (e.g., “Cannot verbalize my feelings well”), Sense of Mindreading Self (e.g., “Am aware of hidden intentions in actions of others”; reverse scoring item), and Sense of Verbal/Narrative Selves (e.g., “Unconsciously tell a lie or a made-up story during conversation”). The internal consistency of the scale was high in the present study (Cronbach’s *α* = 0.88).

##### Independent and interdependent view of self

The independent and interdependent view of self scale (Uchida, 2008) consists of 20 items measured using a 5-point Likert-scale, from 1 (does not describe me at all) to 5 (describes me very much). These items covered two components of the scale: independent view of self (e.g., “I always try to have my own opinions”) and interdependent view of self (e.g., “I am concerned about what people think of me”). Its validity and reliability were verified both in America and Japan (Uchida, 2008). The internal consistency of the independent and view of self scale was high in the present study (Cronbach’s *α* = 0.82). The internal consistency of the interdependent view of self scale was acceptable in the present study (Cronbach’s *α* = 0.70). Preexisting versions of the scales in English and Japanese from past research were used.

##### Dream content and structural dream analysis

Participants answered four items concerning dreams in a free response format to explain the contents of “impressive dreams in childhood,” “recent impressive dreams,” when these dreams occurred, and what happened in reality. For childhood dreams, participants were asked to recall the most memorable dream. As mentioned in the introduction, this study examined participants’ recollections of dreams both from childhood and recently, where all participants wrote about both “impressive dreams during childhood” and “recent impressive dreams.” Given that the time the dreams occurred and what was concurrently happening in their real life were not the focal themes of the current study, participants’ free responses for these items are not included in the current analyzes.

The coding for the free response for these dreams was done using Structural Dream Analysis. Structural Dream Analysis categorizes dreams into the five general structural patterns found in pilot studies (Roesler, 2018b; Roesler et al., 2021). These structures include the relationship between the dream-ego and other figures in the dream, and the extent of agency of the dream-ego, that is, its capacity to act or deal with challenges and the situation of the dream-ego by the end of the dream. Structural Dream Analysis is a qualitative, interpretive research method that attempts to formalize the process of interpretation of the dream in a way that the conclusions are independent from the interpreter.

The five general structural patterns are shown in Table 1. The unit of analysis is the entire dream. One dream can be categorized by more than one pattern. For example, the first half of the dream could be classified as PatternI, but the latter half as Pattern II. Patterns II to V have subclassifications based on the relationship between the dream-ego and others in the dream, the extent of agency of the dream-ego, and the end of the dream. One dream can contain two subclassification from the same variable pattern such as the subclassification of PatternII“The dream-ego is threatened by animals” and “The dream-ego is threatened by human beings” appeared in one dream.

**Table 1:** The seven general structural patterns of structural dream analysis.

**Pattern I “No dream ego present”** : e.g., The dreamer just observes a scene as if watching a movie and does not actively take part in the dream. **Pattern II “The dream-ego is threatened”** : e.g., The dream-ego is threatened, attacked or injured and usually tries to escape or protect itself against the threatening figures. **Pattern III “The dream-ego is confronted with a performance requirement”** : e.g., The dream-ego is confronted with a performance requirement (examination, task to find something which was lost before, or produce something etc.), which is set by another figure or agency in the dream. **Pattern IV “Mobility dream: The dream-ego is moving toward a specified or unclear destination”** : e.g., The dream-ego is moving towards a specified or unclear destination, e.g., traveling and making use of different forms of transportation. **Pattern V “Social interaction dream: The dream-ego is occupied with making contact or communicating with others”** : e.g., The dream-ego wants to get in contact with another figure or attempts to communicate something to the other figure.

## Results

### Comparison of dream descriptives

#### Dream length

Mann–Whitney U test was used to determine the differences in dream length in American and Japanese samples. For “impressive dreams in childhood,” American participants’ dreams (M = 53.02, SD = 37.82) were significantly longer, *U* (*N*_US_ = 220, *N*_Japan_ = 257) = 8884.50, *z* = 12.92, *p <* 0.001, *r* = 0.59, than Japanese participants’ dreams (*M* = 21.09, *SD* = 28.52). In addition, for “recent impressive dreams,” American participants’ dreams (M = 52.36, SD = 49.79) were significantly longer, *U* (*N*_US_ = 220, *N*_Japan_ = 257) = 9381.50, *z* = 12.59, *p <* 0.001, *r* = 0.58, than Japanese participants’ dreams (*M* = 20.65, SD = 33.83).

#### When “impressive dreams in childhood” occurred

Many participants could not recall the exact age the dream occurred and described it “in 〇〇 school days.” Other participants reported their recurring dreams in expressions such as “happened many times in elementary school days.” In addition, participants who reported dreams occurring after senior high school days were removed from the analyzes. Therefore, the data were organized in the following form. In the American sample, 40 dreams occurred during preschool days, 150 dreams occurred during elementary school days, 20 dreams occurred during junior high school days, and 10 dreams occurred during senior high school days. In the Japanese sample, 34 dreams occurred during preschool days, 185 dreams occurred during elementary school days, 30 dreams occurred during junior high school days, and 8 dreams occurred during senior high school days. A Chi-squared test was used to determine the differences in distribution when “impressive dream in childhood” occurred in both samples. Comparisons between American and Japanese samples when “impressive dream in childhood” occurred did not reach significance, *χ*^2^ (3) = 3.52, *p* = 0.319.

#### When “recent impressive dreams” occurred

Many participants could not recall the exact date the dream occurred and described it “〇 weeks/months/year ago” “last week/month/year.” Other participants reported their recurring dreams in expressions such as “happened many times in the past 〇 months/years.” In addition, participants who did not report when their dreams occurred were removed from the analyzes. Therefore, the data were organized in the following form. In the American sample, 71 dreams occurred within a week, 79 dreams occurred within a month, 56 dreams occurred within a year, and 14 dreams occurred within 5 years. In the Japanese sample, 86 dreams occurred within a week, 70 dreams occurred within a month, 82 dreams occurred within a year, and 19 dreams occurred within 5 years. A Chi-squared test was used to determine the differences in distribution when “recent impressive dream” occurred in both samples. Comparisons between American and Japanese samples when “recent impressive dream” occurred did not reach significance, χ^2^ (3) = 4.79, *p* = 0.188.

### Cross-cultural comparisons in self-construal

Each measure for anthropophobia mentality, sense of self, and independent and interdependent view of self was compared between American and Japanese samples using the Welch’s t-test, because the assumption for the equality of variance was not met. The independent view of self was significantly higher in the American sample (*M* = 38.32, SD = 6.36) than in the Japanese sample (*M* = 32.46, SD = 5.30), *t* (427.43) = 10.83, *p* < 0.001, *d* = 1.01. The sense of self was also significantly higher in the American sample (*M* = 92.82, SD = 12.41) than in the Japanese sample (*M* = 85.11, SD = 10.54), *t* (432.16) = 7.25, *p* < 0.001, *d* = 0.67.

On the other hand, the interdependent view of self was significantly lower in the American sample (*M* = 32.88, SD = 6.06) than in the Japanese sample (*M* = 34.68, SD = 4.95), *t* (422.23) = 3.53, *p* < 0.001, *d* = 0.33. Anthropophobia mentality was also significantly lower in the American sample (*M* = 67.04, SD = 45.10) than in the Japanese sample (*M* = 91.67, SD = 37.01), *t* (423.76) = 6.45, *p* < 0.001, *d* = 0.60.

Given that many of these measures are expected to be theoretically related, we also included Tables 2, 3 showing the correlations between the measures in each sample.

**Table 2:** The correlation among each of these measures to one another in the American samples.

Measure	1	2	3	4
1. Anthropophobia mentality	-			
2. Sense of self	−0.59**	-		
3. Independent view of self	−0.56**	0.43**	-	
4. Interdependent view of self	0.38**	−0.18**	−0.32**	-

The significance *p* < 0.01 was marked by **, *p* < 0.05 by *.

**Table 3:** The correlation among each of these measures to one another in the Japanese samples.

Measure	1	2	3	4
1. Anthropophobia mentality	-			
2. Sense of self	−0.50**	-		
3. Independent view of self	−0.38**	0.28**	-	
4. Interdependent view of self	0.15*	−0.00	−0.29**	-

The significance *p* < 0.01 was marked by **, *p* < 0.05 by *.

### Comparison between American and Japanese samples in the dream structure

To reveal whether and how the cultural differences in measures of self-construal reported above reflect the structure of dreams, incidences of “impressive dreams in childhood” and “recent impressive dreams” were compared between American and Japanese samples. A Chi-squared test was used to determine the differences in distribution of dream’s structural patterns in both samples, resulting in 48 multiple comparisons. A Bonferroni-correction was conducted to adjust the significant level for *p* to 0.05/48 to avoid type I error.

#### Impressive dreams in childhood

For “impressive dreams in childhood,” the rate of incidence of each structural pattern in the American and the Japanese samples is shown in Table 4.

**Table 4:** The rate of incidence of each structural pattern in the American and Japanese samples for “impressive dreams in childhood”.

Structural pattern	In American sample	In Japanese sample
PatternI: “There is no dream-ego present”	1 (0.5)	29 (11.3)*
PatternII: “The dream-ego is threatened”	96 (43.6)	95 (37.0)
The subclassification of PatternII: “The dream-ego is damaged, e.g., severely wounded, or even killed. In some cases, the killing has already happened and the dream ego is found as a dead body”	5 (2.3)	16 (6.2)
The subclassification of PatternII: “The threat to the dream ego comes from a force in nature, e.g., a natural disaster, earthquake, fire, flooding, storm etc.”	9 (4.1)	14 (5.4)
The subclassification of PatternII: “The dream-ego is threatened by (dangerous) animals”	24 (10.9)	20 (7.8)
The subclassification of PatternII: “The dream-ego is threatened by human beings, e.g., criminals, murderers or “evil people,” or human-like figures, e.g., ghosts, shadows etc.”	67 (30.5)	54 (21.0)
PatternIII: “The dream-ego is confronted with a performance requirement”	10 (4.5)	20 (7.8)
The subclassification of PatternIII: “Examination in a school or university setting”	1 (0.5)	2 (0.8)
The subclassification of PatternIII: “The dream ego is subject to an inspection by an official person, e.g., a ticket inspection on the train where the right of the dream ego is questioned”	0 (0.0)	1 (0.4)
The subclassification of PatternIII: “The dream-ego has the task to find something (which was lost before), get something, produce something etc.”	9 (4.1)	17 (6.6)
PatternIV: “The dream-ego is moving towards a specified or unclear destination”	95 (43.2)	100 (38.9)
The subclassification of PatternIV: “Disorientation: the dream ego has no idea where to go, even where it is and there are no signs of direction etc.”	17 (7.7)	49 (19.1)*
The subclassification of PatternIV: “The dream-ego is locked up in a closed space, imprisoned etc., and is looking for a way to get out”	3 (1.4)	4 (1.6)
The subclassification of PatternIV: “The dream-ego wants to move, travel etc. but has no means to do so, e.g., it misses the train”	1 (0.5)	2 (0.8)
The subclassification of PatternIV: “The dream-ego attempts to move and has some means of transportation but cannot control the movement, e.g., it cannot steer a car”	6 (2.7)	8 (3.1)
The subclassification of PatternIV: “The dream-ego is moving but the way is blocked or the means of transport breaks down or crashes and movement cannot be continued”	10 (4.5)	3 (1.2)
The subclassification of PatternIV: “The dream-ego is moving, making use of some means of transportation but it is going the wrong way, is in the wrong train or bus, or is not authorized to use it (e.g., has no ticket) and therefore cannot continue the journey”	1 (0.5)	0 (0.0)
The subclassification of PatternIV: “In the positive form, the dream ego succeeds in moving towards and reaching the desired destination”	57 (25.9)*	35 (13.6)
PatternV: “The dream-ego is occupied with making contact or communicating with others”	57 (25.9)	43 (16.7)
The subclassification of PatternV: “The dream-ego wants to get into contact but is ignored by others”	3 (1.4)	6 (2.3)
The subclassification of PatternV: “The dream-ego is criticized, devalued or made ridiculous by others and feels shame”	3 (1.4)	0 (0.0)
The subclassification of PatternV: “The dream-ego is successful in creating the desired contact”	31 (14.1)	17 (6.6)
The subclassification of PatternV: “A special case: the dream ego is aggressive towards others (even kills others) which expresses the will of the dream ego to be separated and autonomous”	12 (5.5)	3 (1.2)
The subclassification of PatternV: “A special case: Positive behavior of others to the dream-ego”	8 (3.6)	17 (6.6)

The American sample contains 220 dreams, and the Japanese sample contains 257 dreams. The table presents the actual numeric value for the number of dreams followed by the rate% in parenthesis for each situation in American and Japanese samples. For example, the rate of the situation that “There is no dream ego present” in American samples is calculated by dividing the number of dreams with the situation that “There is no dream ego present” by the total number of dreams in the American samples. The significance *p* < 0.05 was marked by *. One dream can contain two subclassifications from the same structural pattern. Therefore, the sum of the data number for the subclassification is not equal to the number for the variable pattern.

The rate of the following dream structural patterns was significantly higher in the dreams from the American sample than in the dreams from the Japanese sample; “The dream-ego succeeds in moving toward and reaching the desired destination,” *χ*^2^ (1) = 11.50, *p* = 0.001, *V* = 0.16. Conversely, the rate the following dream structural patterns was significantly higher in the dreams from the Japanese sample than in the dreams from the American sample; “There is no dream-ego present,” *χ*^2^ (1) = 23.59, *p* < 0.001, *V* = 0.22; “The dream-ego has no idea where to go and no sense of direction,” *χ*^2^ (1) = 12.78, *p* < 0.001, *V* = 0.16.

In addition, examples of dreams involving various structural patterns from both the American and Japanese samples are shown in Table 5. The parts of the dreams indicative of the structural patterns are highlighted in italics.

**Table 5:** Examples of the structural patterns identified in the American and the Japanese samples for “impressive dreams in childhood”.

Examples 1–1, 2 of the situation that “The dream-ego succeeds in moving towards and reaching the desired destination” (of IV): in the American sample
“*I remember being in the sky and being able to fly*. I did my best trying to control my flight and seeing the world and *impressing my friends*.”
“I had a dream in which I was an airline pilot. *I traveled around the world and was admired by many*. *It was a nice and pleasant dream I went on many journeys* and leaded a happy life*.”*
Examples 2–1, 2 of the situation that “There is no dream-ego present” (I): in the Japanese sample
*“A big whale was struggling to move thorough big drift ices.”*
“*An elephant squashed my mother.*”
Examples 2–3, 4 of the situation that “the dream-ego has no idea where to go and no sense of direction” (of IV): in the Japanese sample
“My school disappeared, and *I roved where there was no acquaintance such as family or friends*.”
“I cannot forget the dream that *I continued to walk along the endless rail track.*”

In American dreams, there was stronger agency of the dream-ego (Examples 1–1, 2), and higher salience of success of the dream-ego than conflict (Examples 1–1, 2) than in Japanese dreams. On the other hand, in Japanese dreams, the absence or weak agency of the dream-ego was prevalent (Examples 2–1, 2), and vagueness of surroundings and others was also characteristic (Examples 2–3, 4) compared to American dreams. In particular, the distribution of subclassification in the structural pattern “The dream-ego is moving toward a specified or unclear destination” (IV) was apparently different between American and Japanese dreams. In American dreams, the dream-ego has clear will and direction. Whereas, in Japanese dreams, the dream-ego often gets lost or falls for unexplained reasons.

#### Recent impressive dreams

For “recent impressive dreams,” the rate of incidence of each structural pattern in the American and Japanese samples is shown in Table 6.

**Table 6:** The rate of incidence of each general structural pattern in the American and Japanese samples for “recent impressive dreams”.

Situations	In American sample	In Japanese sample
PatternI: “No dream ego present”	1 (0.5)	20 (7.8)*
PatternII: “The dream-ego is threatened”	58 (26.4)	45 (17.5)
The subclassification of PatternII: “The dream-ego is damaged, e.g., severely wounded, or even killed. In some cases the killing has already happened and the dream ego is found as a dead body”	7 (3.2)	9 (3.5)
The subclassification of PatternII: “The threat to the dream ego comes from a force in nature, e.g., a natural disaster, earthquake, fire, flooding, storm etc.”	6 (2.7)	3 (1.2)
The subclassification of PatternII: “The dream-ego is threatened by (dangerous) animals”	8 (3.6)	9 (3.5)
The subclassification of PatternII: “The dream-ego is threatened by human beings, e.g., criminals, murderers or “evil people,” or human-like figures, e.g., ghosts, shadows etc.”	43 (19.5)*	25 (9.7)
PatternIII: “The dream-ego is confronted with a performance requirement”	28 (12.7)	54 (21.0)
The subclassification of PatternIII: “Examination in a school or university setting”	1 (0.5)	0 (0.0)
The subclassification of PatternIII: “The dream ego is subject to an inspection by an official person, e.g., a ticket inspection on the train where the right of the dream ego is questioned”	4 (1.8)	8 (3.1)
The subclassification of PatternIII: “The dream-ego has the task to find something (which was lost before), get something, produce something etc.”	23 (10.5)	46 (17.9)
PatternIV: “The dream-ego is moving towards a specified or unclear destination”	56 (25.5)	43 (16.7)
The subclassification of PatternIV: “Disorientation: the dream ego has no idea where to go, even where it is and there are no signs of direction etc.”	9 (4.1)	8 (3.1)
The subclassification of PatternIV: “The dream-ego is locked up in a closed space, imprisoned etc., and is looking for a way to get out”	4 (1.8)	2 (0.8)
The subclassification of PatternIV: “The dream-ego wants to move, travel etc. but has no means to do so, e.g., it misses the train”	3 (1.4)	1 (0.4)
The subclassification of PatternIV: “The dream-ego attempts to move and has some means of transportation but cannot control the movement, e.g., it cannot steer a car”	9 (4.1)	8 (3.1)
The subclassification of PatternIV: “The dream-ego is moving but the way is blocked or the means of transport breaks down or crashes and movement cannot be continued”	2 (0.9)	4 (1.6)
The subclassification of PatternIV: “The dream-ego is moving, making use of some means of transportation but it is going the wrong way, is in the wrong train or bus, or is not authorized to use it (e.g., has no ticket) and therefore cannot continue the journey”	0 (0.0)	1 (0.4)
The subclassification of PatternIV: “In the positive form, the dream ego succeeds in moving towards and reaching the desired destination”	29 (13.2)	19 (7.4)
PatternV: “The dream-ego is occupied with making contact or communicating with others”	109 (49.5)	95 (37.0)
The subclassification of PatternV: “The dream-ego wants to get into contact but is ignored by others”	13 (5.9)*	1 (0.4)
The subclassification of PatternV: “The dream-ego is criticized, devalued or made ridiculous by others and feels shame”	5 (2.3)	2 (0.8)
The subclassification of PatternV: “The dream-ego is successful in creating the desired contact”	66 (30.0)*	19 (7.4)
The subclassification of PatternV: “A special case: the dream ego is aggressive towards others (even kills others) which expresses the will of the dream ego to be separated and autonomous”	18 (8.2)	15 (5.8)
The subclassification of PatternV: “A special case: Positive behavior of others to the dream-ego”	15 (6.8)	60 (23.3)*

The American sample contains 220 dreams, and the Japanese sample contains 257 dreams. The table presents the actual numeric value for the number of dreams followed by the rate% in parenthesis for each situation in American and Japanese samples. For example, the rate of the situation that “There is no dream ego present” in American samples is calculated by dividing the number of dreams with the situation that “There is no dream ego present” by the total number of dreams in the American samples. The significance *p* < 0.05 was marked by *. One dream can contain two subclassifications from the same structural pattern. Therefore, the sum of the data number for the subclassification is not equal to the number for the variable pattern.

The rate of the following dream structural patterns was significantly higher in the dreams from the American sample than in the dreams from the Japanese sample; “The dream-ego is threatened by human beings,” *χ*^2^ (1) = 9.35, *p* = 0.002, and *V* = 0.14; “The dream-ego wants to get into contact but is ignored by others,” *χ*^2^ (1) = 12.68, *p* < 0.001, and *V* = 0.16; “The dream-ego is successful in creating the desired contact,” *χ*^2^ (1) = 13.44, *p* < 0.001, and *V* = 0.17. The rate of the following dream structural patterns was significantly higher in the dreams from the Japanese sample than in the dreams from the American sample; “There is no dream-ego present,” *χ*^2^ (1) = 15.12, *p* < 0.001, *V* = 0.18; “Positive behavior of others to the dream-ego,” *χ*^2^ (1) = 24.44, *p* < 0.001, and *V* = 0.23.

In addition, examples of dreams involving various structural patterns from the American and the Japanese samples are shown in Table 7. The parts of the dreams indicative of the structural patterns are highlighted in italics. In American dreams, there was stronger agency of the dream-ego (Examples 3–1, 2). Besides, both success (Examples 3–2, 3) and bad results (Examples 3–4) in social interaction happened in American dreams more than in Japanese dreams, which could be regarded as the noted characteristic of American “recent impressive dreams.” On the other hand, in Japanese dreams, the absence or vague agency of the dream-ego was characteristic (Examples 4–1, 2). In addition, the situation where the dream-ego was encouraged by others (Example 4–3, 4) occurred more in Japanese dreams than in American dreams, which could be regarded as the noted characteristic of Japanese “recent impressive dreams.”

**Table 7:** Examples of the structural patterns identified in the American and the Japanese samples for “recent impressive dreams.”

Examples 3–1 of the situation that “The dream-ego is threatened by human beings” (of II): in the American sample
“I was having a shower and then *my home was broken into by a masked man with a gun.* I was able to sneak up behind him and steal the weapon. We struggled and I ended up shooting him. I called the police and attempted to keep him alive while I waited for them to arrive.”
Examples 3–2, 3 of the situation that “The dream-ego is successful in creating the desired contact” (of V): in the American sample
“*I ran for President and won the election.*”
“The most memorable dream I had recently was when I was competing a basketball game. The game was tied, *I took the last shot and made it in. Everyone on my team was happy and celebrated with me winning the game.*”
Examples 3–4 of the situation that “The dream-ego wants to get into contact but is ignored by others” (of V): in the American sample
“I was still with my ex-wife. We were at a party and *she was going to leave with another guy. I was very jealous* and kept trying to convince her to stay with me.”
Examples 4–1, 2 of the situation that “There is no dream-ego present” (I): in the Japanese sample
“*A strange man was bitten by a vampire* and about to be brainwashed. He was desperately trying not to lose consciousness.”
“*A marine beast was flying in the sky*.”
Examples 4–3, 4 of the situation that “Others take the initiative in positive behavior to the dream-ego” (of V): in the Japanese sample
“*I received an old model of “skyline” (car) from a strange friend.* I got a car from someone who was very friendly to me, but I did not know who it was. The ride was also great, but I felt that the position of the front window is high like my body was small.”
“I was admitted to the hospital and taken to the operating room to be anesthetized. I was told that anesthesia would work in an instant, so I was injected with anesthesia with the intention of putting up with it as much as possible. However, *before I knew it, the surgery was over and the doctor told me that “your liver was getting worse but it was okay”*.”

## Discussion

The current research aimed to examine what cultural differences appear between American and Japanese dreams and elucidate the relationship between dream content and self-construal. We hypothesized that the cultural self-construal will be reflected in participants’ dream structures.

The current results revealed the prevalence of the independent view of self in American participants and the interdependent view of self in Japanese participants, corroborating with previous research (Markus and Kitayama, 1991). For Americans, compared to the Japanese, the high sense of self indicating a strong integration of the self may reflect the strength of their ego. This seems to be connected to the characteristically independent view of self where the establishment of the identity is important and valued (Nisbett, 2003). On the other hand, the high anthropophobia mentality for the Japanese compared to Americans may reflect weak agency in interpersonal relationships as well. Whereas low anthropophobia mentality could connect the strong agency on social interaction, which also overlaps with the characteristic of independent view of self.

Based on the results of the cultural self-construal as mentioned above, we offer some interpretation and discussion on the differences in the dream’s structural patterns identified in the American and Japanese samples. The distribution of dream’s structural patterns was significantly different between American and Japanese samples. They differed namely in the relationship between the dream-ego and other figures in the dream, the extent of agency of the dream-ego, and the intensity of other figures or the end of the dream.

As for American dreams, the dream-ego had a clear will and strong mobility such as “The dream-ego succeeds in moving toward and reaching the desired destination” (IV) in “impressive dreams in childhood” or “The dream-ego is successful in creating the desired contact” (V) in “recent impressive dreams.” Notably, in these dream structures, the dream-ego wanted to get admiration or social status was one of the main themes, which reflects that the dream-ego’s strong agency in relationships toward identity establishment is important in American dreams. However, it tends to be confronted by a distinct threat such as “The dream-ego is threatened by human beings” (II) or “The dream-ego wants to get into contact but is ignored by others” (V) in “recent impressive dreams.” These characteristics suggested that identity establishment in American dreams tended to parallel the psychological conflict between the agency of both the dream-ego and others.

For Japanese dreams, the weak agency and vague conscious of the dream-ego were shown in patterns such as “There is no dream-ego present” (I) or “The dream-ego has no idea where to go and no sense of direction” (IV) in “impressive dreams in childhood.” In addition, “There is no dream-ego present” (I) or “Positive behavior of others to the dream-ego” (of VI) in “recent impressive dreams” was characteristic as well. These main structures in Japanese dreams signified that others could play a main role in one’s dreams, which may signify the vague boundary between the dream-ego and others. It was also suggested that relationships with others or sometimes the initiative of others had much importance in Japanese dreams.

Each characteristic of the American and Japanese samples dovetailed with previous research; American-made influence situations evoked stronger feelings of efficacy, whereas Japanese-made adjustment situations evoked stronger feelings of relatedness (Morling et al., 2002). In addition, these characteristics illustrate the nature of traditional culture in the two countries as well. America was a thinly populated and highly fluid area, where frontier mentality and hunting and gathering economic systems were characteristic historically. Therefore, individual independence and assertiveness of one’s ability to others were valued and emphasized in America, which still remains today in the form of the culture of honor (Nisbett, 2003). Whereas, historically, Japan has been an agricultural nation with less mobility due to its farming-related economic system. Such agricultural communities require interdependence and collaboration to flourish, placing importance on social connectedness and relationships and the ability to adjust to roles suitable in the community (Uchida et al., 2019).

In addition, it is possible that the vagueness of the dream-ego’s agency and consciousness in Japanese dreams may affect cultural difference in dream length between American and Japanese dreams. American dreams had obvious ends and a lot of descriptions such as colorful, numerous, and huge. Conversely, the presence of others and the end of Japanese dreams tended to be mundane. In past research, American dreams were also significantly longer than Japanese dreams and the American sample had more frequent lucid dreams (Tsuruta, 2015), which may be in line with the observations in the current study. These differences might be affected by the distinction between the American and Japanese in the independent and interdependent self-construal or our schema of how things work in the world (e.g., ego strength; relationship between conscious and unconscious; narrative format). For instance, in Western people, conscious and unconscious are separated clearly and the ego tends to “unite” the unconscious by “ruling” it (Kawai, 1982). In contrast, in Japanese people, the boundary between conscious and unconscious is “unclear,” so the ego aims to “coexist” with unconscious (Kawai, 1971). This research is novel in validating the use of dream content as implicit measures of self-construal, and can be used to complement other more explicit measures as a means for the measurement of cultural differences.

This article, though, has several methodological limitations. First, the dreams were collected only from America and Japan. Each country from the West or East Asia has both similar and differing aspects in their specific culture or mentality (Nisbett, 2003), so the dreams of non-clinical populations from each country may have differing dream structures. Second, there is the possibility of recollection bias as adults recall their childhood dreams in this research. Moreover, this also opens up the question if this study really measures cultural differences in the recollection of dreams, for both recent and childhood dreams, i.e., everyone dreams the same content, but the type of dreams that gives an impression is culturally influenced. However, one possibility could be that the recollection bias itself is culturally embedded, so it still reflects the differences between America and Japan regarding which types of childhood dreams are memorable to them. Third, the difference in the dream length between America and Japan could affect the current results. For example, it is possible that Japanese are less open/expressive about describing their dreams compared to Americans. Therefore, future studies should be conducted to reveal whether the differences in dream length may influence differences in the amount of actual dream content or just content recalled. Fourth, the current study showed significant differences between American and Japanese samples on anthropophobia mentality, sense of self, and independent and interdependent view of self, without controlling for gender and age, as the violation of the equality of variance assumption limited the validity of regression analyzes. While the findings may not be robust, exploratory analyzes controlling for gender and age did show some differences for the anthoropophobia mentarity, but the general patterns of results remained the same, indicating that these variables should be best controlled for in future studies. Fifth, Structural Dream Analysis is directly applicable for measurements of within-group self-construals, because it focuses on the development of an individual’s psyche. Future studies could use other scales such as Typical Dreams Questionnaire (Nielsen et al., 2003) in combination with Structural Dream Analysis. Typical Dreams Questionnaire aims to identity differences in age, gender, or region and reveal developmental milestones, personality attributes, or sociocultural factors (Nielsen et al., 2003), so the combination of both scales can show the cultural difference in dreams clearer. Besides, in some Japanese dreams, the dream ego moves without clear agency such as this example “I was falling from a height. This dream’s main theme does not fit other dream structures and hence the concept of “Mobility dream” (IV) is most suitable. However, in this dream, the dream ego’s agency seems to be more weak than in the situation that “Disorientation: the dream ego has no idea where to go, even where it is and there are no signs of direction etc.” (of IV). Structural Dream Analysis was derived from German dreams (Roesler, 2018a,b), but additional dream patterns as found in Japanese dreams can be considered for future studies. Sixth, this survey was conducted in June and August 2020, so the spread of COVID-19 may affect the results of this research. Therefore, conclusions drawn by this article should be interpreted carefully.

In addition, given the multicultural background and globalization nowadays, nationality itself may not be the most appropriate representation of culture. Many subcultures may exist among immigrants or between generations of ethnic minorities. While past studies have also used nationality as a proxy for culture (Allen et al., 2014; Eriksson et al., 2019), for greater measurement validity of culture, more differentiation in the ethnicity of the sampled population and other additional measures of culture may be included and controlled for in future research. In addition, the cultural contexts experienced by participants might also change over time. Due to modernization, America and Japan have complex and mixed cultural systems and subgroups with different subcultures, which may not be represented in simplified binary labels for culture. For example, in Japan, there may be a gradual change from traditional cooperative values to put weight on individual’s liberty as influenced by globalization. Unlike Western society which has a long history with religious or philosophical perspectives regarding individualism, in Japan, the compatibility of such new values in a traditionally cooperative society remains a pressing issue (Uchida, 2008). Therefore, future studies could also examine the influence of time period when accessing the development of dreams and self-construal, and compare between specialized sub-groups of immigrants or the second generations of immigrants to discuss the meaning of culture on self-construal and dream-self.

However, the results provide preliminary data for further studies on the connection between dreams and cultural self-construal or the process of self-formation. For instance, each Western country might have both overlapping or distinct aspects of the dream structures. In our separate work examining a German sample, the dream-ego showed a high extent of agency and acted on its own successfully, which resembled the current American sample. However, other structural patterns, such as “The dream-ego wants to get social interactions or statuses,” (of V) did not appear as main dream structures, but “The dream-ego wants to move toward the desired destination on its own” (of IV) was characteristic (Roesler et al., 2021). The tendency found in the German sample was interpreted that “in Germany, maybe even more than in other Western countries, the development of an individualized and autonomous personality with a strong, willful ego and identity is highly valued” (Roesler et al., 2021). Compared to Japan, Western countries are characterized by independent self-construal, but even within these countries, there are different cultural characteristics in terms of ego orientation, which may be reflected in the main dream structure. The same also may apply to East Asian countries for the interdependent self-construal.

In addition, this study suggested that the distinction between American and Japanese samples may reflect the development of an independent or interdependent self which facilitates adaptation to one’s culture. Moreover, there are many psychological symptoms relating to self-construal such as anthropophobia. Therefore, it may be worthwhile to design future research to clarify cultural differences in the connection between the psychological process of self-formation and dream structures. This research showed that the extent of agency of the dream-ego, and the relationship between the dream-ego and other figures, were implicitly influenced by the independent or interdependent self. Within Western or East Asian countries, each characteristic cultural self-construal was found to be reflected in their dreams. Hence, we expand cross-cultural research in introducing a way of establishing identity in each culture based on dream structures.

## Data availability statement

The raw data supporting the conclusions of this article will be made available by the authors, without undue reservation.

## Ethics statement

Written informed consent from the participants was not required to participate in this study in accordance with the national legislation and the institutional requirements.

## Author contributions

HK: conceptualization, methodology, investigation, writing—original draft preparation, and project administration. TK and YT: supervision and validation. HK, TK, YT, and CH: formal analysis. HK, TK, and KB: resources. HK and CH: data curation. TK, CH, KB, and AK: writing—review and editing. HK and AK: visualization. HK and YT: funding acquisition. All authors have read and agreed to the published version of the manuscript.

## Funding

This research was supported by the Uehiro Foundation on Ethics and Education, the IAAP International Association for Analytical Psychology Research Grant, and JSPS KAKENHI Grant Number 19K21816.

## Conflict of interest

The authors declare that the research was conducted in the absence of any commercial or financial relationships that could be construed as a potential conflict of interest.

## Publisher’s note

All claims expressed in this article are solely those of the authors and do not necessarily represent those of their affiliated organizations, or those of the publisher, the editors and the reviewers. Any product that may be evaluated in this article, or claim that may be made by its manufacturer, is not guaranteed or endorsed by the publisher.
